# Chen’s peiyuan tang and premature ovarian failure: unveiling the mechanisms through network pharmacology

**DOI:** 10.3389/fphar.2024.1446707

**Published:** 2024-11-29

**Authors:** Xiao Yang, Yi-Ming Mao, Chong Yao, Ding-ming Song, Yi-bo He, Wei Shen

**Affiliations:** ^1^ The Third Clinical School of Medicine and Rehabilitation School, Zhejiang Chinese Medicine University, Hangzhou, Zhejiang, China; ^2^ Department of Thoracic Surgery, Suzhou Kowloon Hospital, Shanghai Jiao Tong University School of Medicine, Suzhou, Jiangsu, China; ^3^ Huzhou Central Hospital, Fifth School of Clinical Medicine of Zhejiang Chinese Medical University, Huzhou, Zhejiang, China; ^4^ Huzhou Central Hospital, Affiliated Central Hospital of Huzhou University, Huzhou, Zhejiang, China; ^5^ Department of Urology, Jinzhou Medical University, The First Hospital of Jinzhou Medical University, Jinzhou, Liaoning, China; ^6^ Department of Clinical Lab, The First Affiliated Hospital of Zhejiang Chinese Medical University (Zhejiang Provincial Hospital of Chinese Medicine), Hangzhou, Zhejiang, China; ^7^ Pharmacy Compounding Center, The First Affiliated Hospital of Zhejiang Chinese Medical University (Zhejiang Provincial Hospital of Chinese Medicine), Hangzhou, Zhejiang, China

**Keywords:** traditional Chinese medicine (TCM), chen’s peiyuan tang (CSPYT), autophagy, MAPK signaling pathway, network pharmacology

## Abstract

**Background:**

Chen’s Peiyuan Tang (CSPYT) is a compound herbal formula that has shown the potential to enhance ovarian function and reduce autophagy in ovarian granulosa cells, which plays a crucial role in follicular development and maturation. The application of Chinese herbal medicine offers a promising alternative to traditional hormone replacement therapy (HRT).

**Methods:**

This study explores CSPYT’s therapeutic mechanisms in treating POF, focusing on its modulation of autophagy through network pharmacology and transcriptomics-based analysis, predicting potential interactions and pathways. KGN cell line and rat ovarian granulosa cells were used for *in vitro* experiment. 4-Hydroperoxy cyclophosphamide(4-HC) stimulation was carried out for establishing the POF cell model. Q-PCR, Western Blot, Transmission electron microscopy to detect the results.

**Results:**

According to the drug and disease database, the common targets of Chen’s Peiyuan Tang and premature ovarian failure were screened, combined with autophagy gene targets and transcriptome analysis, and finally 8 intersection targets were obtained, namely CDKN1B, MAPK3, PRKCD, CDKN1A, MAPK1, RAF1, BIRC5, CTSB. Enrichment analysis of 8 genes found that they were closely related to the animal autophagy pathway. Construct PPI network diagram. CytoScape 3.9.1 builds CSPYT Drug Target-POF Disease Target-Autophagy Gene Network Diagram. Based on the PPI network diagram and CytoScape 3.9.1 analysis results, it is estimated that MAPK1 and MAPK3 are the key targets of CSPYT in the treatment of POF. The eight final intersection targets were docked with the corresponding active pharmaceutical ingredients. The one that docked most closely with the MAPK family was naringenin. In cell experiment verification, it was confirmed that Chen’s Peiyuan Tang can inhibit the MAPK signaling pathway, significantly reduce the number of autophagosomes, and reduce autophagy damage in ovarian granulosa cells.

**Discussion:**

CSPYT can inhibit the MAPK signaling pathway, prevent autophagy overexpression and restore ovarian granulosa cell function, effectively alleviating the disease pressure of POF.

## Introduction

Premature ovarian failure (POF) is a clinical syndrome that affects women under 40, characterized by amenorrhea, elevated follicle-stimulating hormone (FSH) levels, and diminished estrogen levels, which are accompanied by perimenopausal symptoms such as hot flashes and a reduced libido (Guo et al., 2023). The condition significantly impacts fertility, leading to a decrease in pregnancy and live birth rates and an increased risk of miscarriage, thus adversely affecting the quality of life (Hu S. et al., 2020). Moreover, it is associated with an increased risk of developing cardiovascular disease, osteoporosis, and Alzheimer’s disease in the long term (Tao et al., 2024). Despite its profound health implications, the etiology and pathogenesis of POF are not well understood, and conventional treatments like hormone replacement therapy (HRT) come with potential risks, including the possibility of cancer (Asian institute of clinical oncology aico expert panel, 2023; Xu and Xu, 2023) and recurrence upon discontinuation (Lin et al., 2021). In this context, Traditional Chinese Medicine (TCM) offers a holistic alternative that focuses on restoring ovarian function (Shang et al., 2023).

Central to this alternative approach is Chen’s Peiyuan Tang (CSPYT), a compound herbal formula developed by Professor Chen Xueqi. CSPYT has garnered attention for its potential to enhance ovarian function and mitigate autophagic mechanisms within ovarian granulosa cells, which are critical for follicle development and maturation (Ruan Fei et al., 2022). The formulation’s impact on reducing autophagy suggests a protective role against premature aging and impaired cell function, which are characteristic of POF (Napoletano et al., 2019; Kirkin, 2020). However, the intricate mechanisms by which CSPYT exerts its effects in the context of POF treatment are not fully understood. Given the multi-component and multi-target nature of TCM, elucidating these mechanisms presents a significant challenge. Nevertheless, recent advances in bioinformatics and network pharmacology provide new avenues for dissecting the complex interactions of herbal components and their targets (Cai et al., 2019).

**FIGURE 1:**
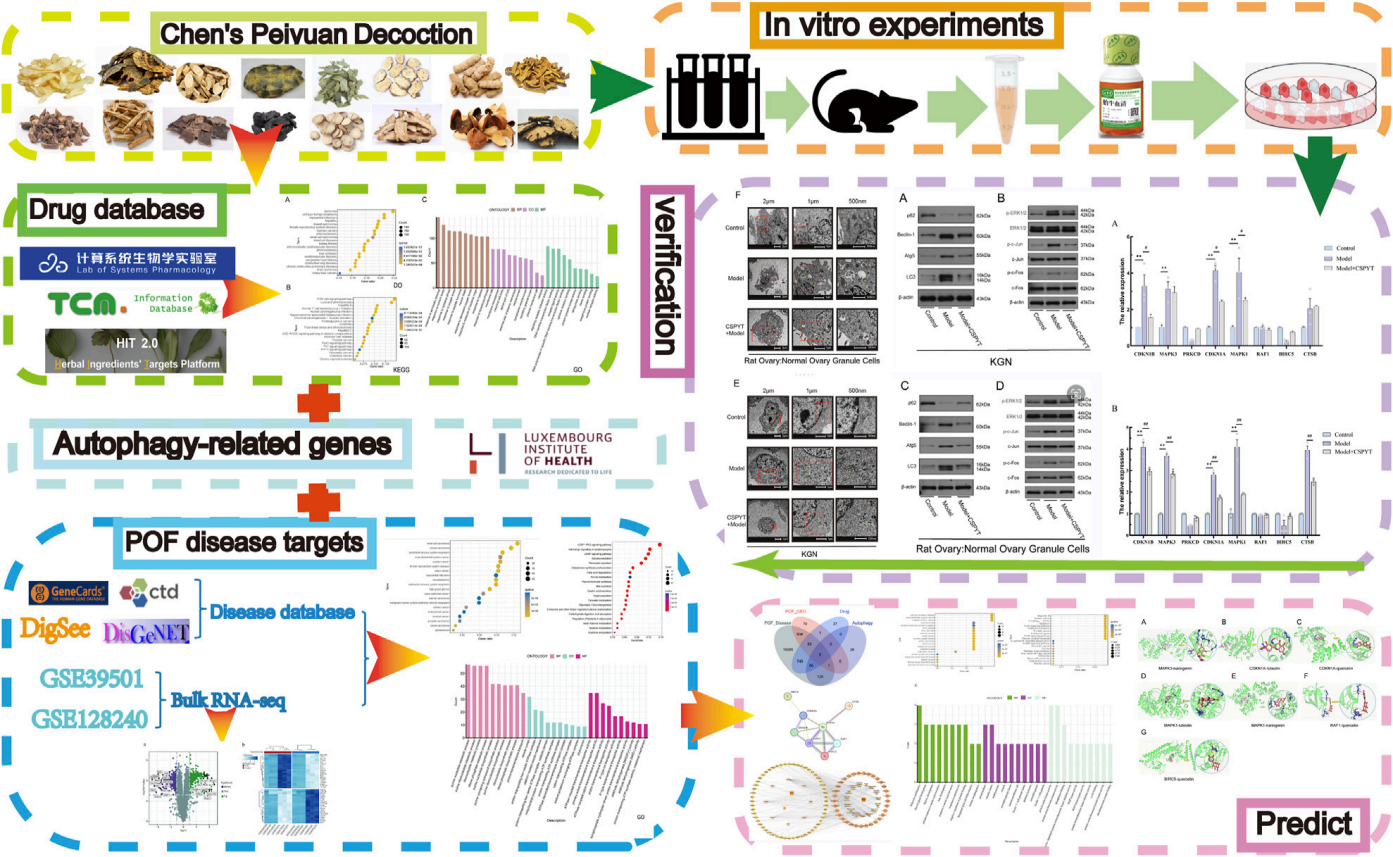
Flow chart of our study.

Despite advancements in understanding POF and its treatment options, the underlying mechanisms of action of TCM formulas like CSPYT remain largely unexplored. While prior studies have suggested that CSPYT may aid ovarian function, this research is novel in employing an integrative approach, using both network pharmacology and transcriptomics to uncover CSPYT’s multi-target effects on autophagy regulation in ovarian granulosa cells. This study is groundbreaking as it provides the first in-depth analysis of CSPYT’s ability to inhibit the MAPK signaling pathway, a critical mechanism for preventing autophagic overactivation in the context of POF. Furthermore, we combine classical cellular experiments with cutting-edge bioinformatics, allowing for comprehensive validation of the pathways and targets predicted by network pharmacology (Zhou et al., 2023). Through this approach, we aim to uncover new insights into CSPYT’s role in ovarian health and establish a basis for future studies on TCM’s potential as an alternative to HRT. By employing network pharmacology and transcriptomics-based analysis, we will predict potential interactions and pathways, which will then be validated through cellular experiments. In this study, cutting-edge science and technology were reasonably used, and the results were comprehensively analyzed in combination with classical experimental methods, which increased the reliability of the conclusions. This comprehensive approach is expected to shed light on the multifaceted effects of CSPYT and its potential as a treatment for POF. Our study process was depicted in Figure 1.

## Methods and materials

### Data preparation

In the meticulous preparation of our data, we identified the active constituents of herbs within the Chinese Spleen-Pancreas-Yin-Tonifying (CSPYT) formulation by consulting three reputable databases: TCMSP (Ru et al., 2014), HIT (Ye et al., 2011), and TCMID (Xue et al., 2012). Our stringent selection criteria were based on compounds with oral bioavailability (OB) ≥ 40% and drug-likeness (DL) ≥ 0.18. The drug targets were then amalgamated from these databases, with duplicates and entries lacking a PubChem ID being meticulously excluded. Subsequently, the corresponding target genes for each active ingredient were delineated using the HIT database, based on the PubChem IDs of the respective compounds.

To address POF, we systematically sourced targets from four established resources, employing the keywords “POF” and “Premature Ovarian Failure.” Our sources included Genecards (Safran et al., 2021), a comprehensive database encompassing genes, their gene products, and their biomedical applications; DisGeNET (Piñero et al., 2017), a repository that consolidates gene-disease associations from various public datasets; DigSee (Kim et al., 2013), a text mining engine that identifies evidence sentences linking genes to diseases through biological events; and CTD (Davis et al., 2023), a robust, publicly accessible database that elucidates the interplay between environmental exposures and human health, featuring curated information on chemical-gene/protein interactions and gene-disease relationships. The disease targets were harmonized from these four databases, with duplicate entries being meticulously expunged.

Additionally, we procured a comprehensive list of autophagy-related genes from the Human Autophagy Database (HADb) (Sullivan et al., 2016), an authoritative public repository that archives information on human genes implicated in the autophagic process. This resource is accessible at http://www.autophagy.lu.

### Transcriptome analysis

In our research, we sourced POF-related high-throughput sequencing datasets from the Gene Expression Omnibus (GEO) database (Du et al., 2023), which is publicly accessible at http://www.ncbi.nlm.nih.gov/geo/. Specifically, we selected two mRNA expression profiling analysis datasets: GSE39501 and GSE128240. The GSE39501 dataset, which is centered on the *Mus musculus* model, encompasses total RNA isolation from various groups including cell transplant recipients (SSCTR), a POF model, and wild type (WT) specimens. This dataset employs microarray technology to discern gene expression characteristics. The GSE128240 dataset, on the other hand, is dedicated to the transcriptome analysis of the impact of cyclophosphamide on mouse ovarian development.

The “combat” method was applied to adjust for batch effects, ensuring the comparability of data across different experimental conditions. Subsequently, the “Limma” R package was employed to systematically screen for differentially expressed genes. Difference analysis was performed with logFC> 0.05, *p* < 0.05 as criteria to screen differential genes (Zhang et al., 2021).

### Network construction and analysis

To delineate the intersections among drug targets, disease targets, transcriptome data, and autophagy genes, Venn diagrams were employed by ‘Venn’ R package. The STRING online tool facilitated the analysis of the final intersecting genes and the generation of protein-protein interaction (PPI) network diagrams. This approach enabled an intuitive examination of complex protein interactions. Network visualization was achieved using the Cytoscape software (Version 3.9.1) to construct a network diagram representing the interplay between CSPYT drug targets, targets of POF, and autophagy-related genes. Key network parameters such as degree, betweenness centrality, and closeness centrality were quantitatively analyzed using the CentiScaPe 2.2 plugin in Cytoscape 3.9.1. These metrics were instrumental in assessing the topological significance of nodes within the network, leading to the identification of hub genes and critical core components.

### Molecular docking

The core genes were screened out according to the Venn diagram, find their corresponding active pharmaceutical ingredients, The inclusion criteria are consistent with the screening of active pharmaceutical ingredients. Use AutoDockTools-1.5.7 for molecular docking, and use Pymol for visual analysis.

### Reagents and cell culture

Human ovarian granulosa cell lines (KGN) and rat ovary normal granulosa cells were obtained from the Cell Resource Center. Fetal bovine serum were procured from Hangzhou Tianhang Biotechnology Co., Ltd. (Hangzhou, China). Rabbit anti-p62 (ab109012, 1:2000, Abcam, Cambridge, United Kingdom), Rabbit anti-Beclin-1 (#3738, 1:2000, CST, Danvers, MA, United States), Rabbit anti-Atg5 (#12994, 1:2000, CST), Rabbit anti-LC3 (#4108, 1:1000, CST), Rabbit anti-p-ERK1/2 (#4370, 1:2000, CST), Rabbit anti-ERK1/2 (#9102, 1:1000, CST), Rabbit anti-p-c-Fos(#5348, 1:1000, CST), Rabbit anti-c-Fos(#31254, 1:1000, CST), Rabbit anti-β-actin (TDY051, 1:10,000, Tiandeyue, Beijing, China), Rabbit anti-p-c-Jun (AF3095, 1:1000, Affinity, Affinity Biosciences, Jiangsu, China), Rabbit anti-c-Jun (AF6090, 1:2000, Affinity), Protease Inhibitor Cocktail (04693159001, ROCHE, Basel, Switzerland), PMSF(AS1006, ASPEN, South Africa), Phosphorylated protease inhibitors (AS1008, ASPEN, South Africa), BCA Protein Assay Kit (AS1086, ASPEN, South Africa), PBS (powder) (AS1025, ASPEN, South Africa), PBS (powder) (AS1025, ASPEN, South Africa), RIPA total protein lysate (AS1004, ASPEN, South Africa), ECL Chemiluminescence Detection Kit (AS1059, ASPEN, South Africa), 5* Protein Loading Buffer (AS1011, ASPEN, South Africa), Osmium acid (80096180, Sinopharm Chemical Reagent Co., Ltd., Shanghai, China), PVDF Membrane (IPVH00010, 0.45 μm, Millipore, United States). TRIpure Total RNA Extraction Reagent (EP013, ELK Biotechnology, Wuhan, China), Electron microscope fixative solution (AS1063, ASPEN Biotechnology CO., LTD., Wuhan, China), 812 Embedding Medium (90,529-77-4, SPI, Beijing, China).

### Preparation of drug-containing serum and cell culture

Twenty female SD rats, the age is 3–7 weeks old, approximately 200 ± 20 g, certified as specifically pathogen-free, were used. Animals were divided into two groups: one receiving oral gavage of saline and the other receiving CSPYT aqueous extract (4 g/ml), The dosage is 0.5 mL/100 g, twice a day. Continuous gavage for 5 days, Intraperitoneal blood was collected 1 hour post-administration on the fifth day, centrifuged, and the supernatant was stored at −80°C.

### Cell culture and experimental modeling

For the cell culture and experimental modeling, we cultured the KGN cell line and rat ovarian granulosa cells in DMEM/F12 medium supplemented with 10% fetal bovine serum and 1% penicillin/streptomycin, maintaining conditions at 37°C with 5% CO_2_. Cells were passaged at approximately 80% confluence using 0.25% trypsin. To establish a model of autophagy or cellular damage, we treated both cell types with 4-Hydroperoxy cyclophosphamide (4-HC) at a concentration of 8 μmol/L for 24 h. This treatment simulates stress conditions that trigger cellular autophagy or damage, which is essential for studying the potential therapeutic effects of treatments or drugs.

### Western blot, RT-PCR and autophagosomes observation

Cells were rinsed with PBS and lysed in a cell protein extraction reagent containing RIPA total protein lysate (pre-added protease inhibitors). After centrifugation at 12,000 g for 5 min at 4°C, the supernatant was collected for protein concentration determination using the BCA Protein Assay kit. Proteins were separated by SDS-PAGE and transferred to a PVDF membrane, which was blocked with 5% skim milk and incubated with primary and secondary antibodies. For electrophoresis, the total protein loading volume of each sample was 40 μg, and an appropriate amount of 5× protein loading buffer was added to the protein sample in a boiling water bath at 95°C-100°C for 5 min. After pouring the membrane, a freshly prepared ECL mixture (A:B = 1:1) was added dropwise to the protein side of the membrane to show the protein bands. Analyzed with AlphaEaseFC software. Total RNA was extracted using TRIpure solution, and cDNA was synthesized and amplified using real-time PCR on a QuantStudio 6 Flex System. The primer gene sequences selected for Q-PCR experiments are listed below (Tables 1, 2).

**TABLE 1:** Human’s Primers sequences of selected genes for q-PCR.

Primer	Base sequence (5′-3`)		Product length
H-ACTIN	sense	GTC​CAC​CGC​AAA​TGC​TTC​TA	190
	antisense	TGC​TGT​CAC​CTT​CAC​CGT​TC	
H-CDKN1B	sense	GGC​TAA​CTC​TGA​GGA​CAC​GCA	115
	antisense	AGA​ATC​GTC​GGT​TGC​AGG​TC	
H-MAPK3	sense	GGA​AGC​CAT​GAG​AGA​TGT​CTA​CAT	111
	antisense	TGG​TAG​AGG​AAG​TAG​CAG​ATA​TGG​T	
H-PRKCD	sense	ATC​AAC​CAG​AAG​CTT​TTG​GCT	111
	antisense	CTT​CTC​GAA​ACC​CTG​ATA​TAT​CC	
H-CDKN1A	sense	CTG​TCA​CTG​TCT​TGT​ACC​CTT​GTG	119
	antisense	TGG​TAG​AAA​TCT​GTC​ATG​CTG​GT	
H-MAPK1	sense	GCT​GAC​TCC​AAA​GCT​CTG​GAC	151
	antisense	CGA​ACT​TGA​ATG​GTG​CTT​CG	
H-RAF1	sense	GTT​GCA​GTA​AAG​ATC​CTA​AAG​GTT	227
	antisense	CAA​TTA​GCT​GGA​ACA​TCT​GAA​ACT​T	
H-BIRC5	sense	TTC​ATC​GTC​GTC​CCT​AGC​CT	210
	antisense	ATC​TCA​GGC​CGA​CTC​AGA​TGT	
H-CTSB	sense	CTA​TGA​ATC​CCA​TGT​AGG​GTG​C	200
	antisense	TGT​CCT​TCT​CGC​TAT​TGG​AGA​C	

**TABLE 2:** Rat’s Primers sequences of selected genes for q-PCR.

Primer	Base sequence (5′-3′)		Product length
R-ACTIN	sense	CGT​TGA​CAT​CCG​TAA​AGA​CCT​C	110
	antisense	TAG​GAG​CCA​GGG​CAG​TAA​TCT	
R-CDKN1B	sense	GAT​ACG​AGT​GGC​AGG​AGG​TG	200
	antisense	GTC​TGA​CGA​GTC​AGG​CAT​TTG	
R-MAPK3	sense	ATG​TCA​TAG​GCA​TCC​GAG​ACA​T	148
	antisense	TAG​AGG​AAG​TAG​CAG​ATG​TGG​TCA​T	
R-PRKCD	sense	CCA​TAA​GAA​ATG​CAT​CGA​CAA​G	130
	antisense	CAT​GTA​GTT​ATA​GAC​CTT​GAA​TCG​G	
R-CDKN1A	sense	CCC​GAG​AAC​GGT​GGA​ACT​T	110
	antisense	CCC​AGG​GCT​CAG​GTA​GAT​CTT	
R-MAPK1	sense	GCA​CCA​ACC​ATT​GAG​CAG​AT	173
	antisense	TCA​CGG​TGC​AGA​ACA​TTA​GCT	
R-RAF1	sense	GTC​ACA​GTG​AAT​CAG​CCT​CAC​C	208
	antisense	GAC​AGC​ATC​ACC​TCA​CTG​GC	
R-BIRC5	sense	CTG​TAC​CTT​AAG​GAC​CAC​CGC	200
	antisense	CTA​TGC​TCC​TCT​ATA​GGG​TTG​TCA​T	
R-CTSB	sense	GCT​GTA​ATG​GTG​GCT​ATC​CCT​C	183
	antisense	TCT​TGT​TGC​ACT​TGG​GAG​TAT​CTC	

Observation of autophagosomes requires fixation, dehydration, embedding, and sectioning of ovarian granulosa cells. Samples were fixed using a transmission electron microscopy (TEM) fixative (Wuhan Aspen Biotech, Cat. No. AS1063). The cells were centrifuged, the culture medium was removed, electron microscope fixative was added, and the cells were fixed at 4°C. The cells were washed four times with PBS, allowing each wash to stand for 15 min. Then 1% osmic acid and 0.1 M phosphate buffer PBS (PH7.4) were added and the cells were fixed for 2 h at room temperature, 20°C. The cells were washed again with PBS four times, with each wash lasting 15 min. Sequential dehydration was performed with 50%, 70%, 80%, 90%, 95%, 100%, and 100% ethanol, each for 15 min. The use of an ethanol gradient for dehydration not only controlled the sample’s dehydration rate but also prevented morphological changes in the sample. The final dehydration was performed twice with 100% ethanol to ensure that all water in the tissue was completely replaced by ethanol. This process ensured more thorough dehydration of the tissue. The ethanol was replaced with acetone twice, allowing each to stand for 15 min. Impregnation was done twice. The first impregnation involved a solution with an acetone-to-embedding agent ratio of 2:1 for 1 h. The second impregnation used a solution with a ratio of 1:2 for 4 h. Two additional impregnations were performed with pure embedding agent, each lasting 24 h. The sample was placed in an embedding plate filled with pure embedding agent and polymerized at 65°C for 48 h. The embedded block was then trimmed into a trapezoidal shape with a surface area of less than 0.2 × 0.2 mm and a section thickness of approximately 70 nm. Images were collected and analyzed using a transmission electron microscope.

### Statistical analysis

A meticulous statistical analysis was conducted utilizing GraphPad Prism 10 (GraphPad Software Inc., California, United States). Continuous variables were meticulously presented as the mean accompanied by the standard error of the mean (SEM). The discernment of statistical disparities among various groups was achieved through the application of a Two-way analysis of variance (ANOVA) followed by the Least Significant Difference (LSD) test. A threshold of *P* < 0.05 was established, thereby deeming any observed discrepancies as statistically significant.

## Results

### Drug target acquisition and enrichment analysis of CSPYT

**FIGURE 2:**
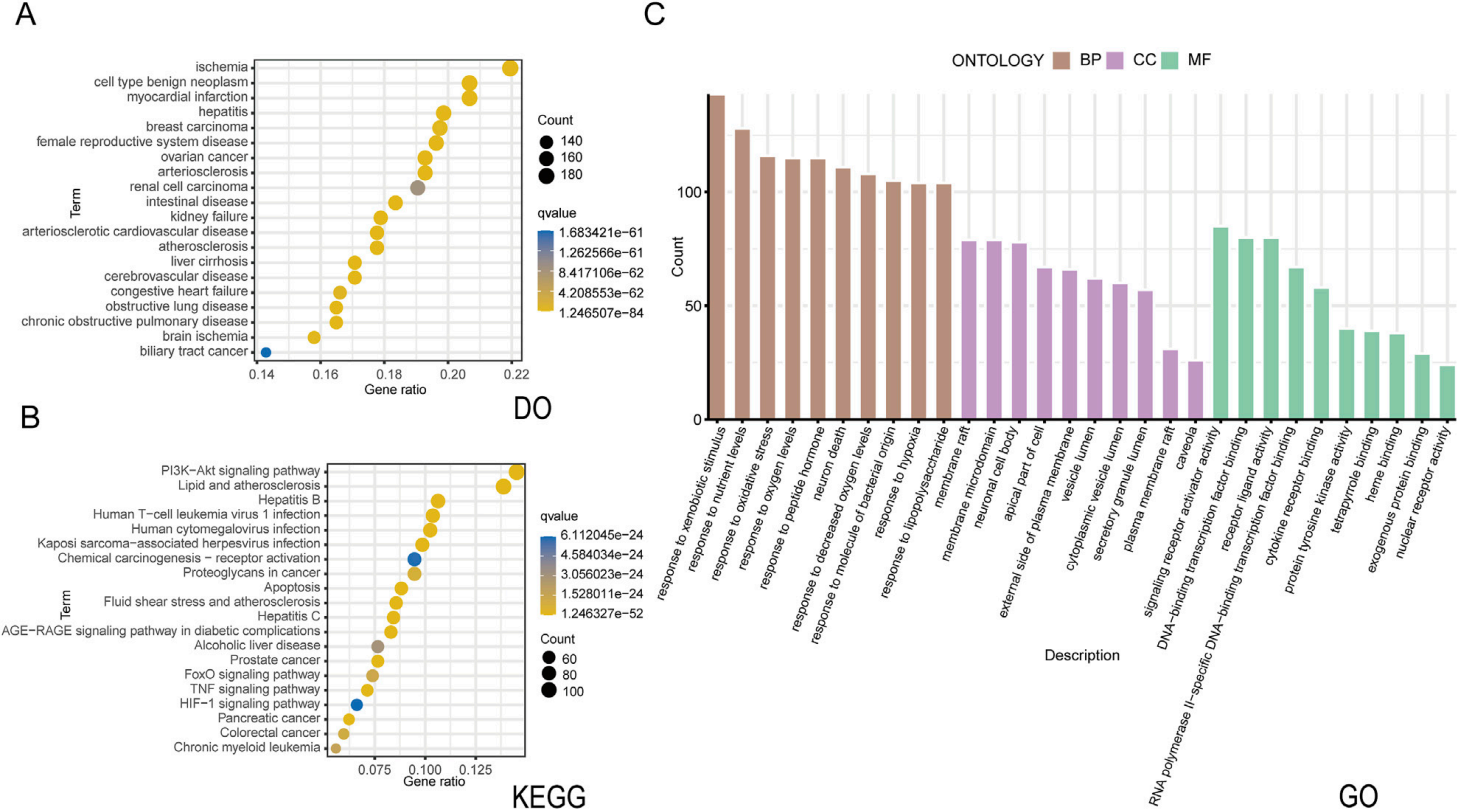
Drug target enrichment analysis of Chen’s Peiyuan Decoction **(A)** The DO analysis of 904 target genes predicted by Chen’s Peiyuan Decoction. **(B)** The KEGG analysis of 904 target genes predicted by Chen’s Peiyuan Decoction. **(C)** The GO analysis of 904 target genes predicted by Chen’s Peiyuan Decoction.

The active constituents of CSPYT, comprising Astragalus membranaceus, Codonopsis radix, Phellodendron chinensis, Radix Chuanxiong, Radix Atractylodes macrocephalus, Polygonatum japonica, Rehmania rehmannia, Paeonia albaeoniae, Fried tangerine peel, Fried Angelica sinensis, Vinegar soft-shelled turtle shell, Xianling spleen, Morinda officinalis, Fried Eucommia ulmoides, and Fried Chuanduan, were meticulously scrutinized across the HIT, TCMSP, and TCMID databases (Table 3). Post-consolidation of the search outcomes and elimination of compounds lacking unique Pubchem IDs or exhibiting redundancy, a total of 1061 active constituents were ascertained. Utilizing the Pubchem ID as a reference, the corresponding target genes for these constituents were identified within the HIT database. After discarding constituents devoid of target gene associations, a collation of the search results yielded 922 target genes linked to 245 active constituents. The QED value, a metric of drug-likeness, was computed for these constituents using the rdkit module in Python, leading to the filtration of 904 target genes associated with 229 potent active constituents based on a QED threshold of >0.2. Enrichment analysis of these drug targets was conducted, with bubble plots employed to elucidate their functional roles, distributions, and pertinent signaling pathways (Figure 2).

**TABLE 3:** Active pharmaceutical ingredient of Chen’s Pei Yuan decoction.

Herb name	MOLID	ingredients	OB (%)	DL
Dangshen	MOL001006	poriferasta-7,22E-dien-3beta-ol	42.98	0.76
	MOL002140	Perlolyrine	65.95	0.27
	MOL002879	Diop	43.59	0.39
	MOL003036	ZINC03978781	43.83	0.76
	MOL000449	Stigmasterol	43.83	0.76
	MOL003896	7-Methoxy-2-methyl isoflavone	42.56	0.20
	MOL004355	Spinasterol	42.98	0.76
	MOL005321	Frutinone A	65.90	0.34
	MOL008397	Daturilin	50.37	0.77
	MOL008400	glycitein	50.48	0.24
	MOL008407	(8S,9S,10R,13R,14S,17R)-17-[(E,2R,5S)-5-ethyl-6-methylhept-3-en-2-yl]-10,13-dimethyl-1,2,4,7,8,9,11,12,14,15,16,17-dodecahydrocyclopenta [a]phenanthren-3-one	45.40	0.76
	MOL008411	11-Hydroxyrankinidine	40.00	0.66
Yinyanghuo`	MOL000098	quercetin	46.43	0.28
	MOL000422	kaempferol	41.88	0.24
	MOL000622	Magnograndiolide	63.71	0.19
	MOL001645	Linoleyl acetate	42.10	0.20
	MOL004367	olivil	62.23	0.41
	MOL004373	Anhydroicaritin	45.41	0.44
	MOL004382	Yinyanghuo A	56.96	0.77
	MOL004384	Yinyanghuo C	45.67	0.50
	MOL004386	Yinyanghuo E	51.63	0.55
	MOL004388	6-hydroxy-11,12-dimethoxy-2,2-dimethyl-1,8-dioxo-2,3,4,8-tetrahydro-1H-isochromeno [3,4-h]isoquinolin-2-ium	60.64	0.66
	MOL004391	8-(3-methylbut-2-enyl)-2-phenyl-chromone	48.54	0.25
	MOL004394	Anhydroicaritin-3-O-alpha-L-rhamnoside	41.58	0.61
	MOL004396	1,2-bis(4-hydroxy-3-methoxyphenyl)propan-1,3-diol	52.31	0.22
	MOL004425	Icariin	41.58	0.61
Yuzhu	MOL000332	n-coumaroyltyramine	85.63	0.20
	MOL000483	(Z)-3-(4-hydroxy-3-methoxy-phenyl)-N-[2-(4-hydroxyphenyl)ethyl]acrylamide	118.35	0.26
	MOL010395	4′,5,7-trihydroxy-6-methyl-8-methoxy-homoisoflavanone	89.70	0.33
	MOL010396	4′,5,7-trihydroxy-6,8-dimethyl-homoisoflavanone	59.76	0.30
	MOL010412	4′-methoxy-5,7-dihydroxy-6,8-dimethyl-homoisflavanone	57.14	0.34
Bajitian	MOL002879	Diop	43.59	0.39
	MOL009495	2-hydroxy-1,5-dimethoxy-6-(methoxymethyl)-9,10-anthraquinone	95.85	0.37
	MOL009496	1,5,7-trihydroxy-6-methoxy-2-methoxymethylanthracenequinone	80.42	0.38
	MOL009500	1,6-dihydroxy-5-methoxy-2-(methoxymethyl)-9,10-anthraquinone	104.54	0.34
	MOL009504	1-hydroxy-6-hydroxymethylanthracenequinone	81.77	0.21
	MOL009513	2-hydroxy-1,8-dimethoxy-7-methoxymethylanthracenequinone	112.30	0.37
	MOL009519	(2R,3S)-(+)-3′,5-Dihydroxy-4,7-dimethoxydihydroflavonol	77.24	0.33
	MOL009537	americanin A	46.71	0.35
	MOL009551	isoprincepin	49.12	0.77
Chenpi	MOL004328	naringenin	59.29	0.21
	MOL005100	5,7-dihydroxy-2-(3-hydroxy-4-methoxyphenyl)chroman-4-one	47.74	0.27
	MOL005815	Citromitin	86.90	0.51
	MOL005828	nobiletin	61.67	0.52
Shudihuang	MOL000449	Stigmasterol	43.83	0.76
Danggui	MOL000449	Stigmasterol	43.83	0.76
Xuduan	MOL003152	Gentisin	64.06	0.21
	MOL009312	(E,E)-3,5-Di-O-caffeoylquinic acid	48.14	0.68
	MOL009323	Sylvestroside III_qt	56.47	0.43
Baishao	MOL001918	paeoniflorgenone	87.59	0.37
	MOL001919	(3S,5R,8R,9R,10S,14S)-3,17-dihydroxy-4,4,8,10,14-pentamethyl-2,3,5,6,7,9-hexahydro-1H-cyclopenta [a]phenanthrene-15,16-dione	43.56	0.53
	MOL001924	paeoniflorin	53.87	0.79
	MOL000211	Mairin	55.38	0.78
	MOL000422	kaempferol	41.88	0.24
	MOL000492	(+)-catechin	54.83	0.24
Chuanxiong	MOL001494	Mandenol	42.00	0.19
	MOL002135	Myricanone	40.60	0.51
	MOL002140	Perlolyrine	65.95	0.27
	MOL002157	wallichilide	42.31	0.71
	MOL000433	FA	68.96	0.71
Duzhong	MOL002058	40,957-99-1	57.20	0.62
	MOL000211	Mairin	55.38	0.78
	MOL000422	kaempferol	41.88	0.24
	MOL004367	olivil	62.23	0.41
	MOL000443	Erythraline	49.18	0.55
	MOL005922	Acanthoside B	43.35	0.77
	MOL006709	AIDS214634	92.43	0.55
	MOL000073	ent-Epicatechin	48.96	0.24
	MOL007563	Yangambin	57.53	0.81
	MOL009009	(+)-medioresinol	87.19	0.62
	MOL009015	(−)-Tabernemontanine	58.67	0.61
	MOL009027	Cyclopamine	55.42	0.82
	MOL009029	Dehydrodiconiferyl alcohol 4,gamma'-di-O-beta-D-glucopyanoside_qt	51.44	0.40
	MOL009031	Cinchonan-9-aL, 6′-methoxy-, (9R)-	68.22	0.40
	MOL009042	Helenalin	77.01	0.19
	MOL009053	4-[(2S,3R)-5-[(E)-3-hydroxyprop-1-enyl]-7-methoxy-3-methylol-2,3-dihydrobenzofuran-2-yl]-2-methoxy-phenol	50.76	0.39
	MOL009055	hirsutin_qt	49.81	0.37
	MOL009057	liriodendrin_qt	53.14	0.80
	MOL000098	quercetin	46.43	0.28
	MOL008240	(E)-3-[4-[(1R,2R)-2-hydroxy-2-(4-hydroxy-3-methoxy-phenyl)-1-methylol-ethoxy]-3-methoxy-phenyl]acrolein	56.32	0.36
Baizhu	MOL000022	14-acetyl-12-senecioyl-2E,8Z,10E-atractylentriol	63.37	0.30
	MOL000049	3β-acetoxyatractylone	54.07	0.22
Huangqi	MOL000211	Mairin	55.38	0.78
	MOL000239	Jaranol	50.83	0.29
	MOL000354	isorhamnetin	49.60	0.31
	MOL000371	3,9-di-O-methylnissolin	53.74	0.48
	MOL000378	7-O-methylisomucronulatol	74.69	0.30
	MOL000380	(6aR,11aR)-9,10-dimethoxy-6a,11a-dihydro-6H-benzofurano [3,2-c]chromen-3-ol	64.26	0.42
	MOL000392	formononetin	69.67	0.21
	MOL000417	Calycosin	47.75	0.24
	MOL000422	kaempferol	41.88	0.24
	MOL000433	FA	68.96	0.71
	MOL000439	isomucronulatol-7,2′-di-O-glucosiole	49.28	0.62
	MOL000098	quercetin	46.43	0.28
Huangbo	MOL002644	Phellopterin	40.19	0.28
	MOL002651	Dehydrotanshinone II A	43.76	0.40
	MOL002662	rutaecarpine	40.30	0.60
	MOL002663	Skimmianin	40.14	0.20
	MOL000449	Stigmasterol	43.83	0.76
	MOL002668	Worenine	45.83	0.87
	MOL000622	Magnograndiolide	63.71	0.19
	MOL000785	palmatine	64.60	0.65
	MOL000787	Fumarine	59.26	0.83
	MOL000098	quercetin	46.43	0.28
	MOL001131	phellamurin_qt	56.60	0.39
	MOL001455	(S)-Canadine	53.83	0.77
	MOL006422	thalifendine	44.41	0.73

### Transcriptome analysis and genetic screening of POF

**FIGURE 3:**
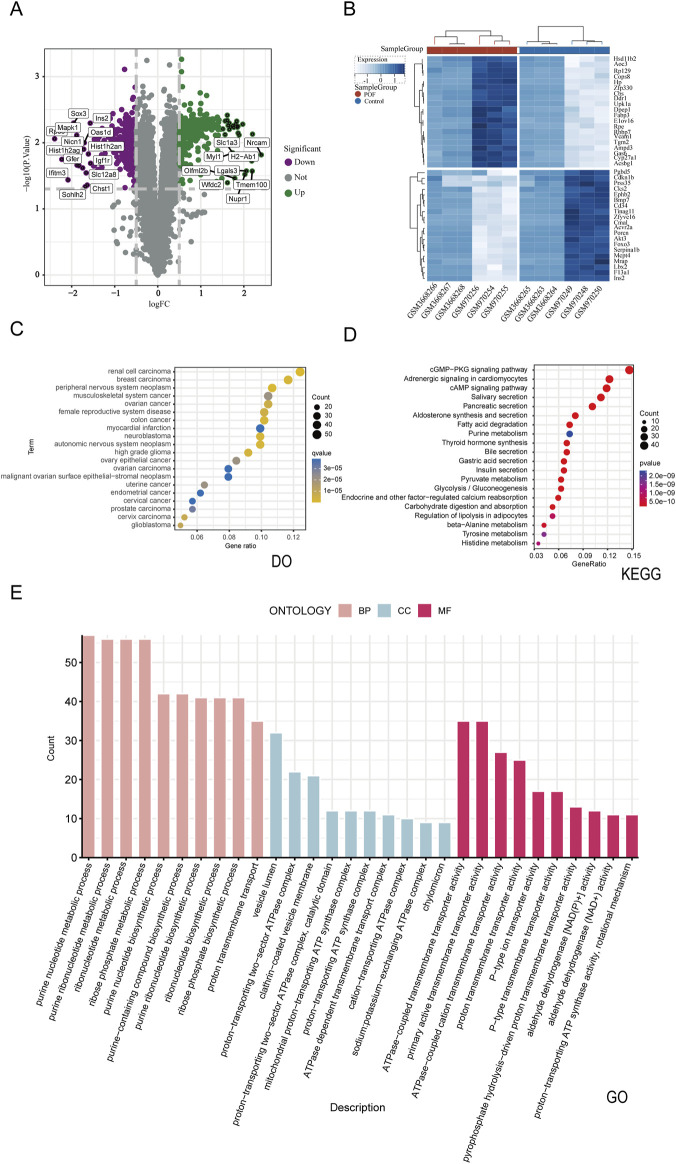
Combining disease database and Bulk RNA-seq transcriptome differential analysis to screen POF disease targets and perform enrichment analysis. **(A)** Volcano plots of differentially expressed genes. The green indicates upregulation,the purple signifies downregulation and the gray represent no significant differences. **(B)** Differentially expressed genes heat map **(C)** The DO enrichment analysis of 566 disease putative targets. **(D)** The KEGG enrichment analysis of 566 disease putative targets. **(E)** The GO enrichment analysis of 566 disease putative targets.

A comprehensive dataset of 2647 POF-related targets was procured from the Genecards database. By evaluating the relevance scores, a median value of 4.09 was established for gene information derived from Genecards, with targets surpassing this threshold deemed as potential POF targets. Integration with DisGeNET, DigSee, and CTD databases facilitated the identification of 20,604 disease-associated genes after deduplication. To enhance the genetic dataset, transcriptome profiles GSE39501 and GSE128240 from the GEO database were incorporated, employing limma for differential gene expression analysis with a stringent filter of *P* value <0.05 and |logFC| >0.5, resulting in the identification of 637 differentially expressed genes (Figures 3A, B). The intersection of these differentially expressed genes with those from the four databases was delineated as the final set of disease-related genes, totaling 566, as visualized by a Venn diagram. Enrichment analysis of these disease targets was performed, with bubble plots utilized to elucidate the target proteins and biological processes implicated in the disease (Figures 3C–E).

### CSPYT-POF-autophagy PPI network analysis

**FIGURE 4:**
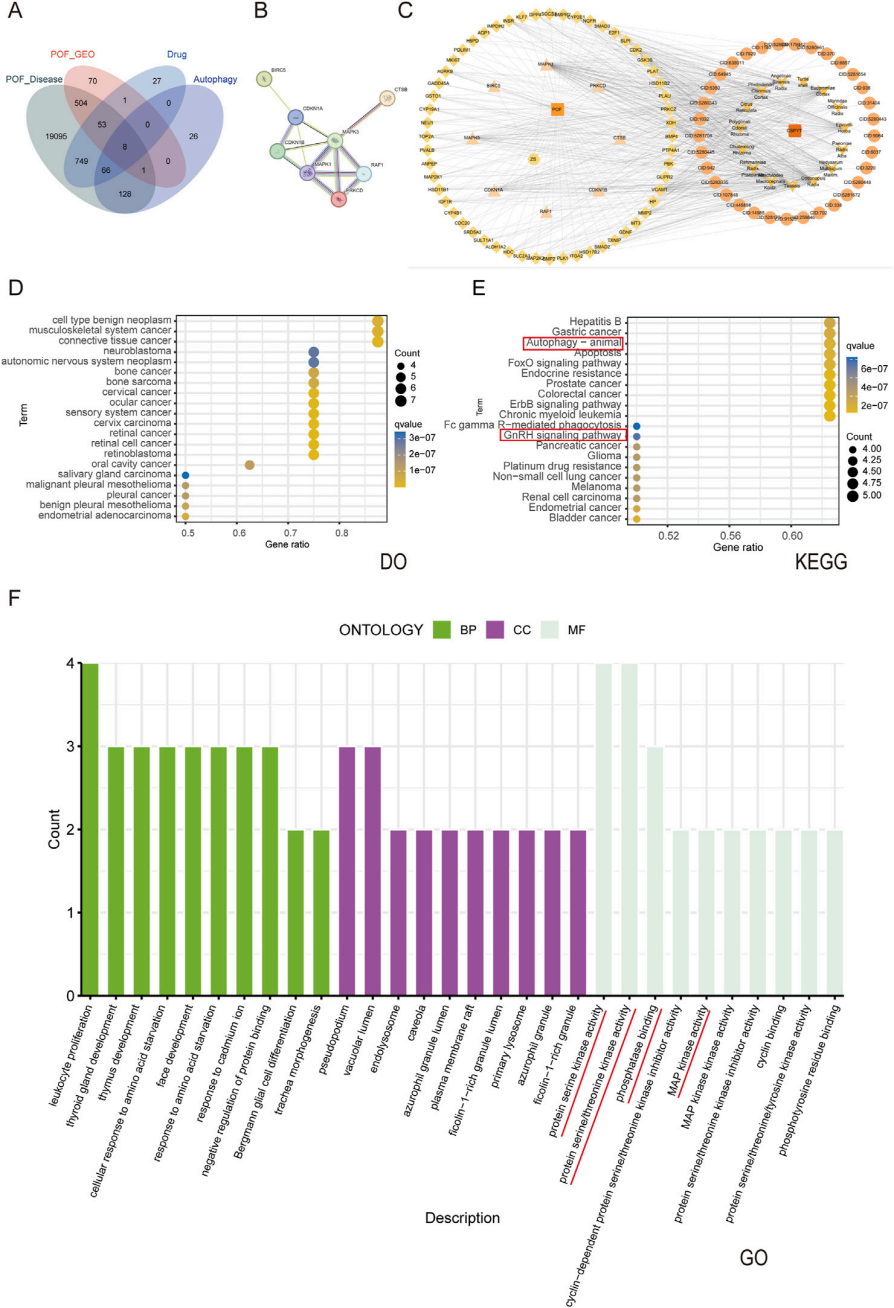
Network analysis of Chen’s Peiyuan Decoction in treating POF through autophagy. **(A)** Venn diagram of 8 genes intersected by Chen’s Peiyuan decoction drug target, POF disease target, GEO database and autophagy gene database. **(B)** The PPI network was plotted for 8 final intersection genes. **(C)** Composition of Chen’s Peiyuan Decoction-target of POF-autophagy network diagram. The arrow is the drug composition of Chen’s Peiyuan Decoction, the circle is the active ingredient, the diamond and triangle is the disease-drug common target, the triangle is the disease-drug-autophagy common target, the CSPYT is the Chen’s Peiyuan Decoction, the POF is premature ovarian failure, and the ZS is autophagy. **(D)** The DO enrichment analysis of 8 intersecting genes. **(E)** The KEGG enrichment analysis of 8 intersecting genes. **(F)** The GO enrichment analysis of 8 intersecting genes.

The convergence of CSPYT drug targets, POF disease targets, GEO transcriptome data, and autophagy-related genes identified 8 core genes (Figure 4A). These core genes were subjected to analysis via the STRING 12.0 database, culminating in the construction of the CSPYT-POF-Autophagy Gene PPI Network, revealing MAPK1 and MAPK3 as the nodes with the highest connectivity (Figure 4B).

### CSPYT for the screening and enrichment analysis of POF and related gene targets

Enrichment analysis of the core genes within the final intersection was executed using R software. KEGG pathway analysis highlighted the involvement of CSPYT in multiple therapeutic targets, including the Autophagy-animals signaling pathway and the GnRH signaling pathway. GO analysis delineated the enriched biological processes, primarily involving responses to chemicals and organic substances, with cellular components localized in the extracellular region, cytoplasm, and plasma membrane. Molecular functions were predominantly linked to protein binding, signal transducer activity, and macromolecular complex binding (Figures 4D–F).

### Construction of CSPYT drug Target-POF disease target-autophagy gene network diagram

The network diagram was constructed using CytoScape 3.9.1 (Figure 4C), with topological parameters analyzed via the CentiScaPe 2.2 Menu. Criteria for core components and hub genes were set at Degree >10.8, Closeness >0.0034, and Betweenness >186.42. Quercetin emerged as the principal active constituent in CSPYT for POF treatment, followed by Ursolic Acid, Myricetin, Vitamin E, and Genistein (Table 4). Among hub genes, CYP2E1 exhibited the highest parameter value, with MAPK1, GSK3B, VCAM1, MAPK3, and CDK2 following (Table 5). Based on CytoScape 3.9.1 analysis and high node genes in the PPI network, MAPK1 and MAPK3, members of the MAPK subfamily, were postulated to be the most intimately associated with CSPYT’s therapeutic effects on POF.

**TABLE 4:** The first five core component parameters obtained by analyzing Chen’s Peiyuan Decoction components-POF-autophagy network topology parameters through the built-in CentiScaPe 2.2 Menu of CytoScape3.9.1.

CID	Drug ingredients	Degree	Closeness	Betweenness
CID:5280343	quercetin	10.40335364	0.00286533	9
CID:64,945	Ursolic Acid	6.113997552	0.002849003	5
CID:5281672	Myricetin	3.786492758	0.002754821	4
CID:14,985	Vitamin E	2.656252596	0.002770083	3
CID:5280961	Genistein	2.223619846	0.00265252	3

**TABLE 5:** The hub genes parameters obtained by analyzing Chen’s Peiyuan Decoction components-POF-autophagy network topology parameters through the built-in CentiScaPe 2.2 Menu of CytoScape3.9.1.

Gene	Degree	Closeness	Betweenness
CYP2E1	380.1432882	0.003937008	17
MAPK1	334.3909083	0.004065041	75
GSK3B	253.9693341	0.003846154	17
VCAM1	234.9219777	0.003968254	29
MAPK3	208.8377213	0.004	27
CDK2	188.1464992	0.004	23

### Molecular docking of key targets

**FIGURE 5:**
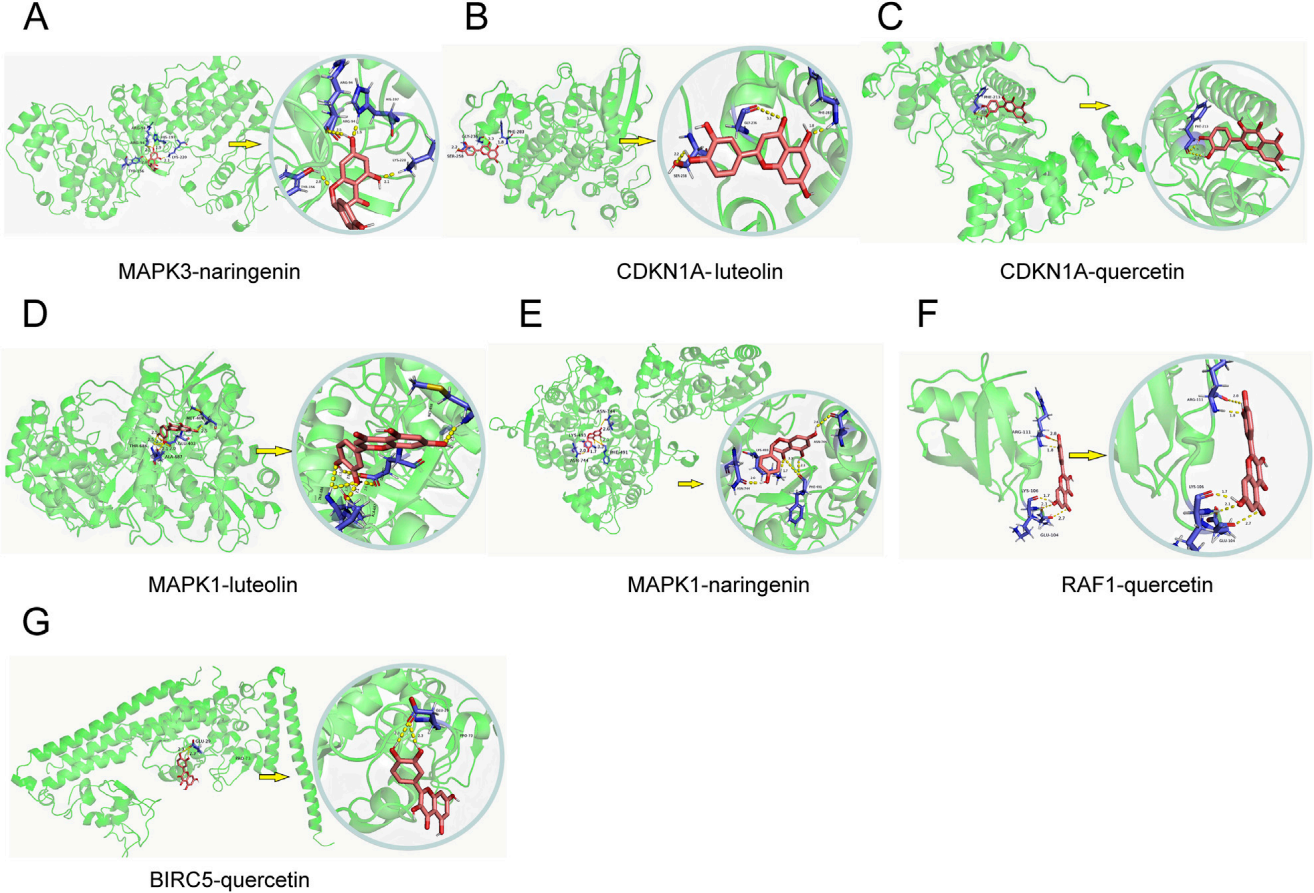
Molecular docking results. **(A–G)** Molecular docking of 8 core targets with corresponding drug active ingredients.

Molecular docking was conducted for the active pharmaceutical ingredients corresponding to the key genes, yielding binding energies post-docking. The selection of three core components (quercetin, luteolin, naringenin) for docking with the core targets of MAPK3, CDKN1A, MAPK1, RAF1, and BIRC5 (Figure 5) demonstrated spontaneous binding in the natural state, as indicated by negative binding energies (<0 kcalmol−1), affirming the strong interaction between these proteins and small molecules (Table 6).

**TABLE 6:** Molecular docking results of the eight final intersection genes and the corresponding active ingredients of Chen’s Peiyuan Decoction.

Gene Symbol	Entry	PDB ID	MOL ID	Drug ingredients	Binding energy (kcal/mol)
MAPK3	P27361	4QTB	MOL004328	naringenin	−3.23
CDKN1A	P38936	1BLX	MOL000006	luteolin	−3.78
CDKN1A	P38936	1BLX	MOL000098	quercetin	−1.04
MAPK1	P28482	4LZ5	MOL000006	luteolin	−2.43
MAPK1	P28482	4LZ5	MOL004328	naringenin	−1.93
RAF1	P04049	1RFA	MOL000098	quercetin	−1.38
BIRC5	O15392	2RAX	MOL000098	quercetin	−1.31

#### CSPYT enhances autophagy in ovarian granulosa cells via the MAPK pathway

**FIGURE 6:**
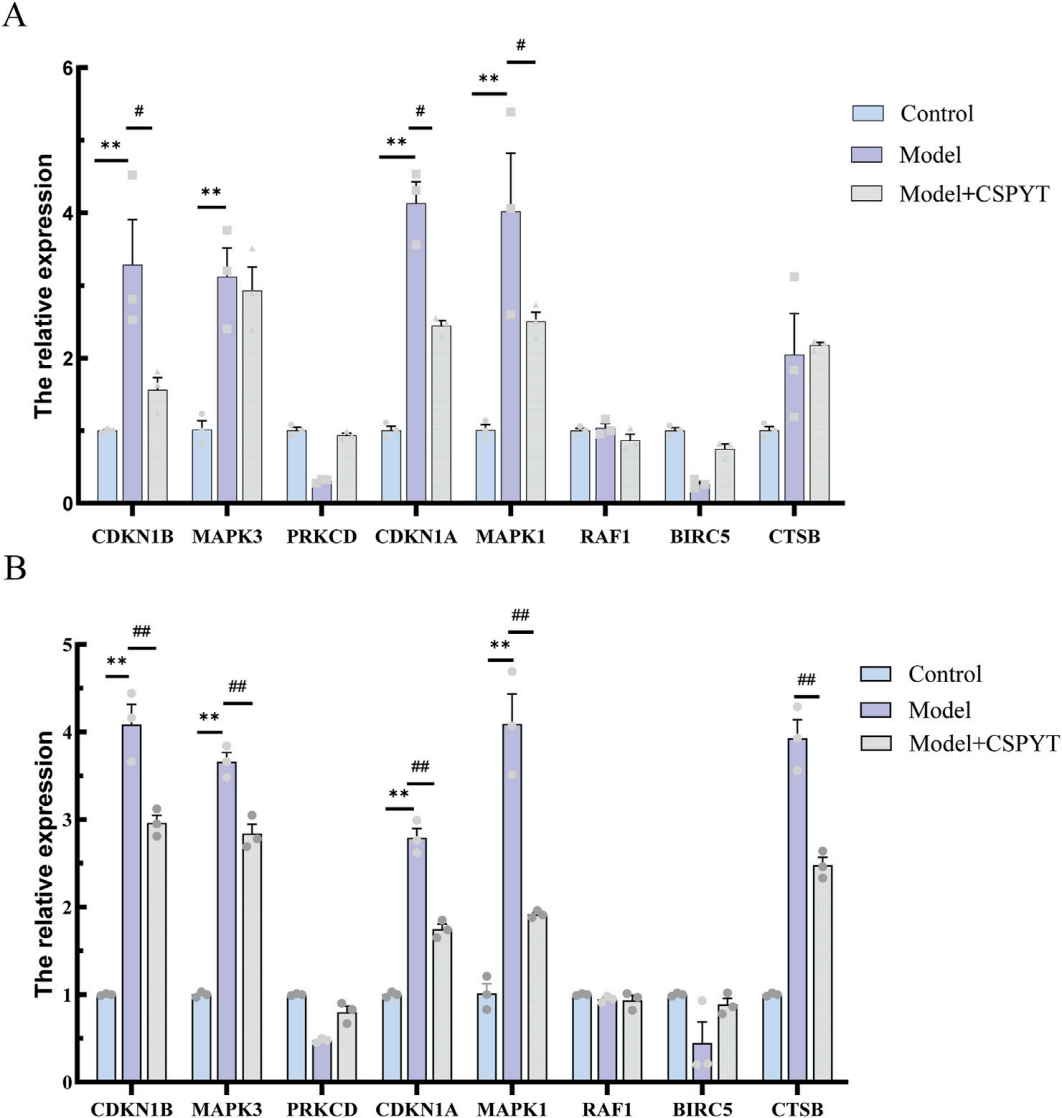
Detection of the 8 core targets using q-PCR in two cell lines:KGN and RatOvary:NormalOvaryGranuleCells. **(A)** KGN test results. **(B)** Rat Ovary:Normal Ovary Granule Cells test results. KGN and Rat Ovary:Normal Ovary Granule Cells exposed to physiological saline, cyclosporine, and cyclosporine plus Chen’s Peiyuan Decoction medicated serum. All data are expressed as mean ± SEM for three samples per group.*P < 0.001 vs. control group,**P < 0.0001 vs. control group,# *P* < 0.001 vs. Model group,## *P* < 0.0001 vs. Model group.

*In vitro* validation of network pharmacology predictions was conducted through q-PCR quantification of eight genes, including CDKN1B, MAPK3, PRKCD, CDKN1A, MAPK1, RAF1, BIRC5, and CTSB. RNA extraction from cell lysates preceded q-PCR analysis, revealing a significant upregulation of all genes except PRKCD and BIRC5 following 4-HC-induced autophagy. The addition of CSPYT-containing serum to the model group resulted in a downregulation of CDKN1B, CDKN1A, MAPK1, and MAPK3 expressions in both KGN cell lines and Rat Ovary: Normal Ovary Granule cell lines, with statistical significance (Figure 6).

### Detection of autophagy-related proteins via WB

**FIGURE 7:**
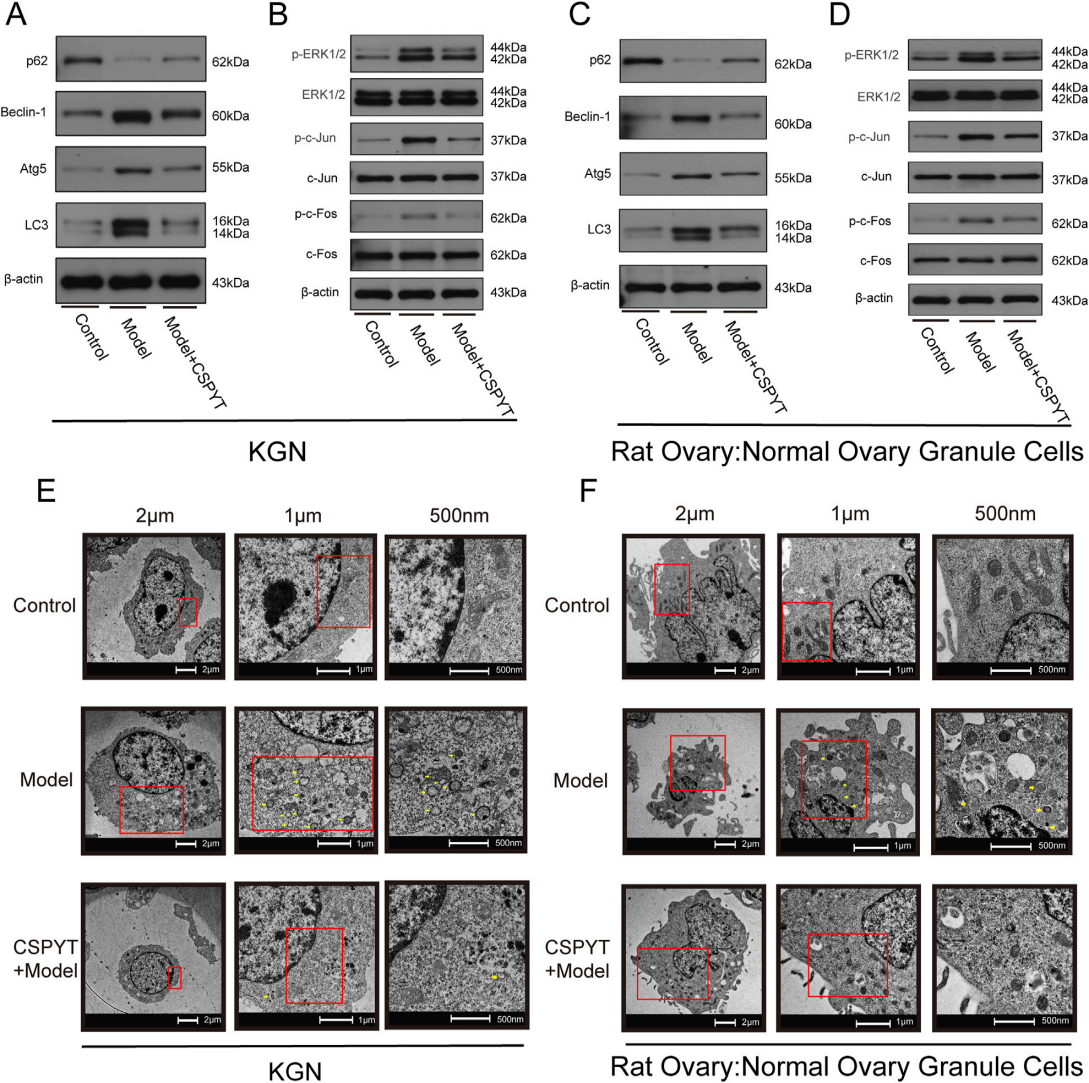
*In vitro* experimental verification of Chen’s Peiyuan Decoction in treating POF through autophagy. **(A–F)** KGN and Rat Ovary:Normal Ovary Granule Cells exposed to physiological saline, cyclosporine, and cyclosporine plus Chen’s Peiyuan Decoction medicated serum. **(A)** Representative western blot images of the relative expressions of p62,Beclin1,LC3, and Atg5 in each group of KGN. **(B)** Representative western blot images of the relative expressions of p-ERK1/2,ERK1/2,LC3,p-c-Jun,c-Jun,p-c-Fos and c-Fos in each group of KGN. **(C)** Representative western blot images of the relative expressions of p62,Beclin1,LC3,and Atg5 in each group of Rat Ovary:Normal Ovary Granule Cells. **(D)** Representative western blot images of the relative expressions of p-ERK1/2,ERK1/2,LC3,p-c-Jun,c-Jun,p-c-Fos and c-Fos in each group of Rat Ovary:Normal Ovary Granule Cells. **(E)** Observation of autophagosomes in each group in KGN by TEM. **(F)** Observation of autophagosomes in each group in Rat Ovary: Normal Ovary Granule Cells by TEM.

Western Blot analysis of autophagy-related proteins indicated a significant downregulation of P62 and upregulation of Beclin-1, Atg5, and LC3 in the model group compared to the Control group. The addition of CSPYT-containing serum modulated these expressions, with P62 levels increasing and Beclin-1, Atg5, and LC3 levels decreasing. Focusing on the MAPK signaling pathway, which was identified as the most pertinent to autophagy regulation, downstream phosphorylation sites were assessed. The model group exhibited elevated levels of p-ERK1/2 (Ma and Nicolet, 2023), p-c-Jun (Zhang et al., 2018), and p-c-Fos (Yokoyama et al., 2013), indicative of MAPK pathway activation, which was subsequently inhibited by the addition of CSPYT-containing serum, suggesting the ability of CSPYT to impede the MAPK pathway (Figures 7A–D).

### TEM observation of autophagosomes

Transmission electron microscopy revealed a marked increase in autophagosome presence in the model group compared to the control, with notable formation of autophagolysosomes due to endomembrane lysis. The administration of CSPYT-containing serum to the model group resulted in a significant reduction in autophagosome numbers and a normalization of cytoplasmic organization (Figures 7E, F).

## Discussion

In recent years, the incidence of POF has continued to rise, showing a concerning trend towards younger individuals. The current standard treatment is HRT (Sullivan et al., 2016), but its long-term use is associated with an increased risk of breast (Xu and Xu, 2023), endometrial, bladder cancer (Wang et al., 2020), and ovarian cancers. A single treatment modality cannot fully match the living conditions of different populations. Diverse drugs that can be more adapted to each patient’s own situation are urgently needed. Mesenchymal stem cell differentiation therapy and other means are also used to treat POF (He et al., 2022). Embryo and ovarian tissue freezing are commonly used methods to preserve the fertility of young patients with POF, but these methods are both risky and costly (Cartwright et al., 2010). Therefore, developing new therapeutic recommendations for POF patients is a pressing challenge.

TCM has shown considerable promise in clinical practice. However, due to the multi-component, multi-target, and multi-pathway characteristics of TCM formulas, understanding their mechanisms of action is challenging. With advancements in science and technology, network pharmacology has emerged as a valuable tool for investigating the mechanisms of TCM. CSPYT, a classic prescription by Professor Chen Xueqi for treating POF, has been observed to effectively alleviate symptoms caused by autonomic disorders in POF patients, such as hot flashes, night sweats, and mood disturbances. Previous animal studies have indicated that CSPYT can significantly reduce the production of autophagosomes and regulate autophagy-related proteins in rat POF models. However, its mechanism of action remains unclear. In this study, we used network pharmacology and *in vitro* experiments to elucidate CSPYT’s mechanism in regulating POF through autophagy.

In the network pharmacology analysis, CSPYT targets, disease targets (from the GEO database) (Li et al., 2023), and autophagy targets were screened through public databases, identifying eight intersecting genes: CDKN1B, MAPK3, PRKCD, CDKN1A, MAPK1, RAF1, BIRC5, and CTSB. These core genes are central to our study. Functional enrichment analysis of these core genes revealed that CSPYT regulates multiple signaling pathways, such as autophagy, apoptosis, and oxidative stress. At the molecular functional level, these pathways are mainly related to protein serine/threonine/tyrosine kinase activity, protein serine/threonine kinase inhibitor activity, phosphatase binding, MAP kinase activity, and MAP kinase activity. The MAPK pathway, a tertiary kinase cascade, includes MAP kinase kinase (MKKK), MAP kinase (MKK), and MAPK, activated sequentially. Serine/threonine protein kinases catalyze the phosphorylation of serine or threonine residues on target proteins using ATP as a phosphate donor. Serine/threonine protein kinases include cyclin-dependent kinases, mitogen-activated protein kinases (MAPKs), protein kinase D, nattokinase, DNA-dependent protein kinase, Aurora protein kinases, and pancreatic kininogenase (Pearson et al., 2001). We hypothesize that CSPYT regulation involves MAPKs expression. MAPKs are not only serine/threonine protein kinases but also key regulators of autophagy, capable of bidirectional regulation (Samare-Najaf et al., 2023). Using the Cytoscape CentiScaPe 2.2 Menu, we analyzed the CSPYT drug ingredient-POF disease target-autophagy target network diagram to identify hub genes, including CYP2E1, MAPK1, GSK3B, VCAM1, MAPK3, and CDK2. MAPK1 and MAPK3 coincided with the core genes, suggesting that MAPK is a critical target for CSPYT in treating POF through autophagy, primarily through phosphorylation activation of signaling pathways.

Key active ingredients in CSPYT, identified through cytoscape analysis, include quercetin. Quercetin significantly increased AMH, LH, and FSH levels and upregulated their receptors in CTX-induced POF mice (Zheng et al., 2022). By increasing AMH protein and receptor levels, quercetin reversed ovarian dysfunction and increased primordial follicles. Additionally, increased levels of FSH, LH, and their receptors promoted follicular development. Similarly, in a study by Chen et al., quercetin significantly increased AMH, E2, and P levels, reducing follicle degradation (Chen et al., 2022). Improved ovarian functional markers and morphology indicated quercetin’s therapeutic potential for POF (Jian et al., 2024). Ursolic acid, another active ingredient, has antioxidant, anti-inflammatory, and anti-tumor effects, regulating NF-κB and MAPK signaling pathways to reduce pro-inflammatory factors and increase anti-inflammatory factors. Ursolic acid’s strong antioxidant capacity can alleviate oxidative stress in immune-related POF caused by autophagy (Limami et al., 2023). Genistein, have excellent pharmacological activities against osteoporosis, cardiovascular diseases, sexual dysfunction, inflammation, and cancers (Lu et al., 2022), also an important active ingredient, attenuates apoptosis by upregulating ER-β and increasing FOXL2 expression, which downregulates TGF-β, inhibiting ovarian follicle transformation and retaining primordial follicles (Haddad et al., 2020). Naringenin, a flavanone, showed strong anti-inflammatory and antioxidant activities, activating the MAPK signaling pathway, suggesting it as CSPYT’s primary active ingredient for treating POF through autophagy (Naraki et al., 2021).

Regardless of the cause, POF ultimately disrupts the HPO gonadal axis, causing hormonal disorders, follicular atresia, or premature follicular depletion, leading to reduced follicular reserves. In short, the function of the ovaries is showing the phenomenon of aging within the visual range of people (Shen et al., 2022). At the same time, it has also become a risk factor for cancer (Chen et al., 2024). At the microscopic level, the aging of ovarian function is associated with apoptosis of ovarian granulosa cells. Granulosa cell apoptosis directly causes follicular atresia, and autophagy plays a critical role in this process. As an intracellular recycling mechanism, autophagy has a dual role in regulating ovarian granulosa cells. Under normal conditions, autophagy maintains cell homeostasis (Wang et al., 2023), but its overactivation or inhibition disrupts homeostasis and function (Liang et al., 2022). Beclin-1 regulates autophagosome maturation, and LC3, once activated, transforms into LC3Ⅱ, a fat-soluble autophagy marker. p62, bridging LC3 and ubiquitination, promotes autophagic degradation and is phagocytosed by autophagosomes, decreasing its levels. Atg5 promotes LC3A to LC3B conversion (Wang et al., 2022). Our results showed downregulated p62 and upregulated Beclin-1, Atg5, and LC3B, indicating enhanced autophagy. *In vitro* q-PCR results showed significantly increased MAPK expression in the model group compared to controls, which was relatively decreased in the model + CSPYT group, suggesting CSPYT inhibited MAPK expression. WB experiment results showed increased autophagic flux in the model group and activation of MAPK downstream proteins, whereas the model + CSPYT group showed relatively decreased autophagic flux and inhibition of MAPK downstream proteins. Thus, CSPYT treats POF by inhibiting the MAPK signaling pathway and preventing autophagy overexpression.

Although alternative treatments for POF include several options across both Western medicine and TCM. Western medicine options, are primarily limited to HRT. HRT is effective in alleviating menopausal symptoms and reducing the risks of osteoporosis. However, it carries an increased risk of hormone-sensitive cancers (Asian institute of clinical oncology aico expert panel, 2023), such as breast and ovarian cancers, making it unsuitable for long-term use in some patients (Sullivan et al., 2016). The suitability of estrogen replacement or estrogen-pregnancy mixture replacement for different age groups remains unconfirmed and requires more updated evidence (Lubo, 2017). When it comes to TCM, there are promising options like Kuntai Capsules and Ziyin Mixture. These herbal treatments work by modulating endocrine function, reducing oxidative stress, and restoring ovarian health. Studies have shown that Kuntai Capsules can improve ovarian reserve markers and reduce oxidative damage, benefiting overall ovarian function (Lin et al., 2021). Similarly, Ziyin Mixture, known for its nourishing and replenishing properties, has been shown to elevate estrogen levels and support menstrual health, helping manage symptoms of POF. Additionally, phytoestrogens (such as those in soy isoflavones) and DHEA supplementation serve as supportive treatments. Phytoestrogens mimic estrogen by binding to estrogen receptors, providing a weaker but similar effect that helps manage symptoms like hot flashes and supports bone health. Meanwhile, DHEA acts as a precursor to sex hormones, potentially enhancing ovarian function by increasing estrogen and testosterone levels in women with diminished ovarian reserves (Haddad et al., 2020). However, popular market drugs often fall short in meeting the diverse needs of all patients due to the unique and varied constitutions of individuals. Consequently, traditional Chinese medicine (TCM) emphasizes “syndrome differentiation and treatment”, ensuring that prescriptions are adaptable and varied to meet individual needs. CSPYT, in particular, stands out as a critical component in this approach for the management of POF. Systematic research is validating CSPYT’s efficacy and developing various prescriptions to cater to unique patient constitutions. CSPYT’s importance lies in its potential to offer highly personalized treatment options, tailored to individual patients. With ongoing research and development, CSPYT holds promise for providing more comprehensive and effective treatments in the future.

We explored the therapeutic potential of CSPYT in the treatment of POF, with a focus on its ability to regulate autophagy and restore ovarian granulosa cell function. However, several limitations of this research should be acknowledged. First, the study was primarily conducted using *in vitro* models, which, while valuable for elucidating cellular mechanisms, cannot fully replicate the complex physiological environment of a living organism. Future research should employ *in vivo* animal models to further validate the efficacy and safety of CSPYT in more physiologically relevant settings. Additionally, although network pharmacology is a powerful tool for predicting drug-target interactions, it relies heavily on existing databases, which may not comprehensively cover all potential biological interactions. Further experimental validation of these predicted targets is essential. Moreover, while key active components of CSPYT, such as naringenin and quercetin, were identified, the study did not test these components independently, which limits our understanding of their individual contributions to CSPYT’s overall therapeutic effect. Although the multi-component, synergistic approach of TCM was followed, future studies should also investigate the specific roles of individual components. Furthermore, the lack of clinical data is a significant limitation. Clinical trials will be necessary to confirm the safety and efficacy of CSPYT in POF patients and to compare its effectiveness with existing treatments such as HRT. Lastly, POF is a heterogeneous condition with various etiologies, and the therapeutic response to CSPYT may differ depending on the underlying cause. Future research should aim to investigate how different subtypes of POF respond to CSPYT to optimize personalized treatment strategies. These limitations provide important directions for future research and further clinical application of CSPYT. A limitation of our study is that we did not test the independent effects of individual components, such as naringenin, on autophagy and granulosa cell function. While our focus was on the synergistic effects of CSPYT as a whole formula, future studies should explore the specific contributions of key compounds like naringenin. This limitation has been addressed in the Discussion section as a potential direction for future research.

Our study highlights the therapeutic potential of CSPYT in treating POF by regulating autophagy and inhibiting the MAPK signaling pathway. However, further research is needed to fully understand its mechanisms and clinical applicability. Future studies should focus on *in vivo* validation using animal models to better simulate human physiological environments, as current findings are primarily based on *in vitro* models (Tao et al., 2024). It is also crucial to investigate the individual contributions of active ingredients such as naringenin and quercetin to clarify their specific roles in CSPYT’s therapeutic effects (Shang et al., 2023). Moreover, clinical trials are essential to assess CSPYT’s safety and effectiveness as an alternative to HRT, especially given the risks associated with long-term HRT use, such as cancer (Guo et al., 2023). Given the heterogeneous nature of POF, personalized treatment strategies should also be explored to improve outcomes for different subtypes of patients (Hu S. B. et al., 2020). Furthermore, while network pharmacology provided insights into CSPYT’s potential targets, it is limited by existing databases. Expanding these databases and integrating advanced experimental methods will enhance the accuracy of target predictions, supporting CSPYT’s integration into clinical practice (Shang et al., 2023). Ultimately, TCM-based therapies like CSPYT offer a promising alternative for treating POF, not only addressing fertility concerns but also mitigating long-term health risks, though further validation is required to bring this therapy closer to clinical use.

## Conclusion

This study investigated CSPYT’s mechanism on POF using network pharmacology and *in vitro* experiments. We found that naringenin, CSPYT’s main active ingredient, controls autophagy overactivation and restores ovarian granulosa cell function by inhibiting the MAPK signaling pathway.

This study was only verified by cell experiments, and it is different from the complex physiological internal environment of humans. Animal experiments can better simulate human disease states, and it is hoped that animal experiments will be conducted in the future to further clarify the mechanism of action. However, the environment in which humans live is complex, and the transmission of diseases is complex. A single model analogy cannot bridge the gap between human physiology and pathology. Although network pharmacology can roughly screen the active ingredients of drugs, it cannot accurately locate them. This is the future. A major research challenge.

## Data Availability

The datasets presented in this study can be found in online repositories. The names of the repository/repositories and accession number(s) can be found in the article/supplementary material.

## Glossary

CSPYT: Chen’s Peiyuan Tang
POF: Premature Ovarian Failure
TCM: Traditional Chinese Medicine
HRT: hormone replacement therapy
TCMSP: Traditional Chinese Medicine Systems Pharmacology Database and Analysis Platform
HIT: Herbal Ingredients’ Targets Platform
TCMID: Traditional Chinese Medicine Integrated Database
CTD: The Comparative Toxicogenomics Database
HADb: Human Autophagy Database
GEO: Gene Expression Omnibus
DL: drug-likeness
OB: oral bioavailability
PPI: protein-protein interaction
GO: gene Ontology DO:disease Ontology
KEGG: Kyoto Encyclopedia of Genes and Genomes
KGN: human ovarian granulosa cell lines
SSCTR: cell transplant recipients
WT: wild type
4-HC: hydroperoxy cyclophosphamide
WB: western blot
RT-PCR: Reverse Transcription-Polymerase Chain Reactionm
TEM: transmission electron microscopy
SEM: standard error of the mean
ANOVA: analysis of variance
LSD: Least Significant Difference
QED: quantitative estimate of drug-likeness
MAPK: mitogen-activated protein kinase
CTX: cyclophosphamide
FSH: follicle-stimulating hormone
LH: load half word
AMH: anti-Müllerian hormone
E2: estradiol
P: progesterone
HPO: hypothalamic-pituitary-ovarian
